# The “Trinity” smart hospital construction policy promotes the development of hospitals and health management in China

**DOI:** 10.3389/fpubh.2023.1219407

**Published:** 2023-07-21

**Authors:** Guang-Wei Zhang, Mengchun Gong, Hui-Jun Li, Shuang Wang, Da-Xin Gong

**Affiliations:** ^1^Department of Smart Hospital Management, The First Hospital of China Medical University, Shenyang, China; ^2^The Internet Hospital of the First Hospital of China Medical University, Shenyang, China; ^3^The Internet Hospital Branch of the Chinese Research Hospital Association, Beijing, China; ^4^Digital Health China Co. Ltd., Beijing, China; ^5^Shenyang Medical & Film Science and Technology Co. Ltd., Shenyang, China; ^6^Enduring Medicine Smart Innovation Research Institute, Shenyang, China; ^7^Department of General Practice, The First Hospital of China Medical University, Shenyang, China

**Keywords:** Trinity, smart hospital, smart medicine, smart services, smart management

## Abstract

Recently, in order to comprehensively promote the development of medical institutions and solve the nationwide problems in the healthcare fields, the government of China developed an innovative national policy of “Trinity” smart hospital construction, which includes “smart medicine,” “smart services,” and “smart management”. The prototype of the evaluation system has been established, and a large number of construction achievements have emerged in many hospitals. In this article, the summary of this field was performed to provide a reference for medical workers, managers of hospitals, and policymakers.

## Introduction

**Figure 1:**
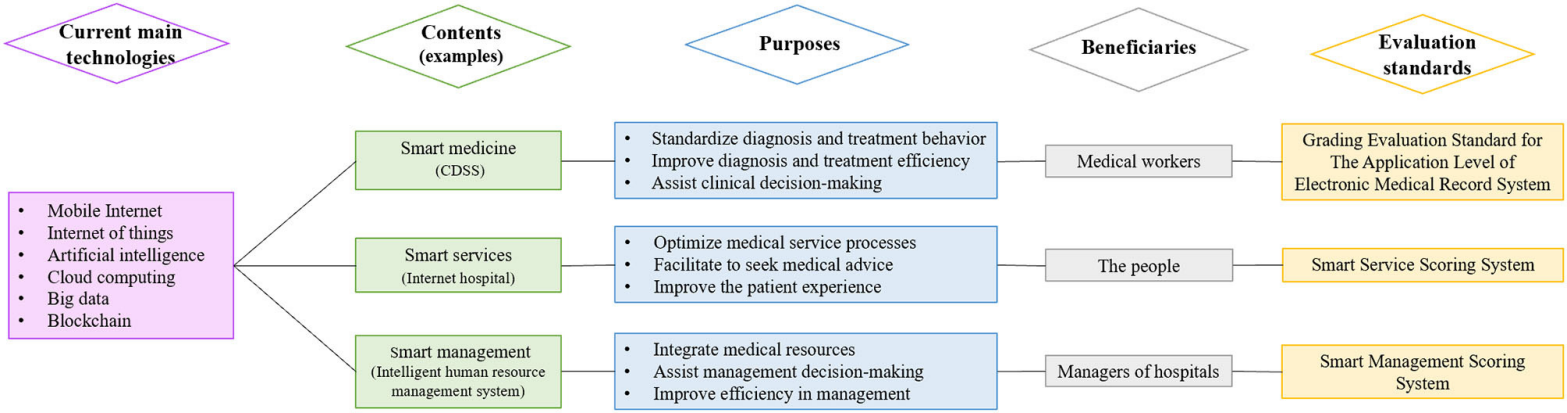
Diagram of the relationship between contents, purposes, beneficiaries, and evaluation standards of “Trinity” smart hospital construction. CDSS, Clinical decision support system.

“Trinity” smart hospital construction, an innovative national policy developed by China's government, has emerged as strategically significant in the healthcare field. This new policy aimed to address the projections of the increasing population needs for healthcare services, increasing gap coverage of regions with barriers to healthcare services accessibility, and increasing the quality of healthcare services (1). As shown in Figure 1, smart hospital construction refers to the process of integrating medical resources, optimizing medical service processes, standardizing diagnosis and treatment behavior, improving diagnosis and treatment efficiency, assisting clinical and management decision-making, and realizing the convenience of seeking medical advice, as well as refining the management by using advanced technologies [such as mobile Internet, Internet of things, artificial intelligence (AI), cloud computing, and big data]. “Trinity” includes “smart medicine” for medical workers, “smart services” for people, and “smart management” for managers, whereby the integration of the three tenets is central to the future development of hospitals.

For these aspects, the respective evaluation standards have been issued with Chinese documents on the National Health Commission of the People's Republic of China's website. First, the “Grading Evaluation Standard for The Application Level of Electronic Medical Record System” was used to assess the supporting ability of electronic medical record systems to clinical behavior through functions, effective application, technical basic environment, and data quality (2). Second, the “Smart Service Scoring System” aimed to evaluate service ability supported by information system for patients with the following 17 items, namely, visit appointment, emergency connection, referral service, service information push, guidepost and navigation, convenience of basic support services, pathway of patient feedback, chronic disease management, drug dispensing and distribution, family doctor services, guidance and education for grassroots physician, convenience of expenses payment, intelligent medical guidance, health education, telemedicine, information security management, and service supervision (3). Third, the “Smart Management Scoring System” was formulated for the assessment of management ability supported by information system through the following 10 items, namely, security of information system, medical care, human resource, financial asset, equipment and facilities, drug consumables, operational, logistics supplement, teaching and scientific research, and office management (4).

Additionally, for further enhancing the implementation of the “Trinity” policy, various government departments have also issued a series of policy-related documents to encourage hospitals to explore and innovate by using advanced intelligence technology. For example, the opinions of the general office of the State Council on promoting the development of “Internet + medical health” aimed to promote Internet applications in healthcare fields (5). “Notice of the State Council on printing and distributing the development plan of the new generation of AI” was issued for the promotion of medical AI applications (6). “Smart hospital construction guide” has been published by local governments on the website of the National Public Service platform to further refine the construction and evaluation standards (7). These evaluation standards and guiding documents not only constitute the “Trinity” smart hospital evaluation system but also become important guidelines for specific construction objectives.

To implement the national policy on the construction of “Trinity” smart hospitals, many hospitals have begun to increase investments in human, material, and financial resources and even set up independent full-time departments in recent years. In these hospitals, benefiting from advanced construction concepts and active application of new technologies, historical phased progress has emerged in medical treatment, service, and management. The specific performances included the increase in work efficiency and accuracy, the improvement of patient satisfaction and outcomes, and the enhancement of management potency, which have comprehensively improved the high-quality development of hospitals (1, 8–11). For example, the tiered medical systems in China have promoted inter-hospital medical information interconnection and mutual recognition of results and consequently brought significant benefits in improving the efficiency of medical treatment and avoiding repeated examinations (12). Additionally, hospital–community–family-based healthcare systems have, indeed, improved the level of regional off-hospital healthcare management, especially in self-management (13). The Internet Hospital of Zhongshan Ophthalmic Center of Sun Yat-sen University provided a non-contact virtual clinical service for patients with eye diseases during the COVID-19 outbreak, avoiding the risk of transmission during medical visits (14). Some other typical cases are exemplary as shown on the website of the National Health Commission of the People's Republic of China (15), including the 5G+augmented reality technique remote emergency system and full process mobile intelligence medical service system developed by Sir Run Run Shaw Hospital of Zhejiang University School of Medicine, the unified and interactive information integration platform inside and outside the hospital built by Guangdong Women and Children Hospital, and the supercomputing center dominating the construction of smart hospital designed by Beijing Tiantan Hospital. These achievements have strongly proven the effectiveness of this policy and indicated its feasibility of long-term implementation.

There is a wealth of global evidence confirming that hospital digitalization indeed leads to improved work efficiency, enhanced healthcare quality, and strengthened collaboration and communication (16, 17). As reported by Smart et al., online consultation within South West England effectively mitigated the workload and stress of healthcare professionals during the COVID-19 pandemic (18). It is revealed in a multinational study that ophthalmologists were more willing to use AI as clinical assistive tools, indicating that hospital digitalization has received widespread acceptance and support from doctors (19). In addition, there has been a further increase in the likelihood of successful implementation of the “Trinity” smart hospital in China. Furthermore, drawing upon our practical experience at The First Hospital of China Medical University, it is essential to harness the proactive engagement of medical staff in order to facilitate their active involvement and initiative.

Compared with HIMSS AEMRA, a worldwide-used standard to evaluate the informatization level of medical institutions (20), the “Trinity” smart hospital scoring system encourages medical information systems to realize intelligent functions with the help of new technologies. Additionally, it puts more emphasis on the following three aspects: (1) the balanced development of hospitals in medical treatment, service, and management, (2) the inter-hospital information interconnection and the process of service, and (3) the response potential for major public health events such as COVID-19 pandemic (10). Therefore, it may be more suitable for China's national conditions and is greatly expected to promote the development of medical institutions and the reform of the medical industry.

In our opinion, as a new concept generated in the process of information technology development meeting the needs of the medical industry, the “Trinity” smart hospital may need to be gradually understood, accepted, and implemented. Its development and improvement really depend on a lot of practical support. Additionally, there is no definite end with final standards for this process because the continuous iteration of technology will keep driving the goals of construction higher and higher. The present summary is expected to provide a reference for medical workers, managers of hospitals, and policymakers.

## Author contributions

G-WZ and D-XG contributed to the manuscript opinions and wrote the manuscript. H-JL helped with manuscript preparation. MG and SW provided critical feedback on the manuscript. All authors have read and approved the content of the manuscript.
